# Species identification and mitochondrial genomes of ancient fish bones from the Riverine Kachemak tradition of the Kenai Peninsula, Alaska

**DOI:** 10.1080/23802359.2018.1456371

**Published:** 2018-04-01

**Authors:** Alida de Flamingh, Elizabeth K. Mallott, Alfred L. Roca, Alan S. Boraas, Ripan S. Malhi

**Affiliations:** a Program in Ecology, Evolution and Conservation Biology, University of Illinois at Urbana-Champaign, Urbana, IL, USA;; b Department of Anthropology, Northwestern University, Evanston, IL, USA;; c Carl R. Woese Institute for Genomic Biology, University of Illinois at Urbana-Champaign, Urbana, IL, USA;; d Department of Animal Sciences, University of Illinois at Urbana-Champaign, Urbana, IL, USA;; e Department of Anthropology, University of Alaska, Anchorage, AK, USA;; f Department of Anthropology, University of Illinois at Urbana-Champaign, Urbana, IL, USA

**Keywords:** Ancient DNA, salmon, sockeye mitogenome, coho mitogenome

## Abstract

Seven fish vertebrae were chosen for analysis from the 49-KEN-147 archaeological site in the Kenai Peninsula, Alaska. Mitochondrial DNA analysis of the ancient fish bones revealed that they were from sockeye and coho salmon. Here, we report the ancient mitochondrial genomes for three sockeye salmon and one coho salmon fish bone.

Seven fish vertebrae were chosen for analysis from the 49-KEN-147 archaeological site (60°28′53.98″N, 151°7′17.96″W; Reger 1998) in the Kenai Peninsula, Alaska. The bones are from a within-house conical storage pit (50 cm × 30 cm) of the Riverine Kachemak tradition that contained 1166 ribs, fins, and vertebrae. Wood structural material from the house dated between 1890 ± 60 and 1570 ± 30 radiocarbon years while the fish bones dated considerably older at 2520 ± 30 radiocarbon years. The discrepancy between the terrestrial dates of wood and the anadromous salmon dates could be due to marine reservoir effect although the almost 1000-year difference is much larger than previously reported for the North Pacific (Edinborough et al. 2016). Ancient DNA analysis provides an effective way of determining the species identity of each of these fish bones (Grier et al. 2013). Here, we used molecular techniques to extract DNA and sequence whole mitochondrial genomes for these samples.

DNA extractions were carried out in a dedicated clean room at the ancient DNA laboratory in the Carl R. Woese Institute for Genomic Biology of the University of Illinois at Urbana-Champaign (UIUC) following a protocol optimized for ancient DNA extraction (Cui et al. 2013). Unused fishbone fragments and extracted DNA samples have been placed in permanent storage in the ancient DNA laboratory (Carl R. Woese Institute for Genomic Biology, Urbana, IL). DNA extracts were tested for endogenous fish DNA by amplifying two short DNA sequences from the cytochrome b (CYTB) and D-loop regions on the mitochondrial genome. These primers have previously been used to identify different fish species from bones at archaeological sites (Yang et al. 2004). We were able to amplify the CYTB region for six and the D-loop region for all seven fish bones (GenBank accession numbers: CYTB MG993156–MG993161, D-loop MG993149–MG993155). We compared these sequences to previously reported sequences on GenBank using basic local alignment search tool (Altschul et al. 1990). The CYTB region for five of the six sequences and the D-loop region for six of the seven sequences matched sockeye salmon (*Oncorhynchus nerka*). A single fish bone had CYTB and D-loop sequences that matched coho salmon (*Oncorhynchus kisutch*). All but one of the corresponding GenBank sequences were exact matches (100% identity) for the entire fish bone sequence (100% coverage). One D-loop sequence that matched sockeye salmon had a 99% identity for 100% coverage due to a single indel.

To reconstruct the mitochondrial genome, we selected four samples that had distinct haplotypes for the short mtDNA regions: three of the sockeye salmon and the single coho salmon. We used a NEBNext Ultra DNA Library Prep Kit for Illumina in combination with NEBNext single index primers to build genomic libraries for each of these fish bones. Libraries were pooled and submitted for HiSeq 4000 sequencing at the UIUC Core Sequencing Facility, which produced over 215 million reads for the pool.

Reads were de-multiplexed and sequences were trimmed using AdapterRemoval (Lindgreen 2012) with the minimum sequence length set to 25 bp. Trimmed sequences were aligned to the *Oncorhynchus kisutch* and *Oncorhynchus nerka* mitogenomes (NC009263 and NC008615, respectively) using bowtie2 (Langmead and Salzberg 2012) with the local alignment option and capping fragment length at 1000 bp. Aligned sequences were transformed to BAM format in SAMtools v. 1.1 (Li et al. 2009), removing unmapped reads and reads with a quality score less than 30. Aligned files were sorted and indexed using SAMtools v. 1.1 and PCR duplicates were removed in SAMtools v. 0.1.19 (Li et al. 2009). Consensus sequences were generated from the de-duplicated alignment files in Geneious R7 (www.geneious.com). Variants were called in SNVer (Wei et al. 2011) with a mapping quality of 30, a base quality of 20, an alternate/reference ratio of 0.9, and an alternate threshold count of 200. Ancient DNA damage patterns were verified by aligning trimmed reads to the *O. kisutch* or *O. nerka* mitogenomes with BWA (Li and Durbin 2010) and quantifying damage in mapDamage2 (Jónsson et al. 2013).

A maximum likelihood (ML) phylogeny was inferred using RaXML v. 8 (Stamatakis 2014); it included mitogenomes from the four fish bones and 10 previously published reference genomes (accession numbers provided in Figure 1) for all North American salmon species, which included Pacific salmon (chinook, chum, coho, pink, and sockeye) and Atlantic salmon. Sequences were aligned using Geneious R7 and the ML tree was drawn using FigTreeV 1.4.2 (Rambaut and Drummond 2009). ML analysis parameters included 1000 bootstrap pseudoreplicates and the GTR + Gamma evolutionary model. The ML analysis supported the results of the short mtDNA regions, as three of the mitogenomes grouped with previously reported sockeye salmon and the fourth mitogenome with a previously reported coho salmon mitogenome.

**Figure 1.**
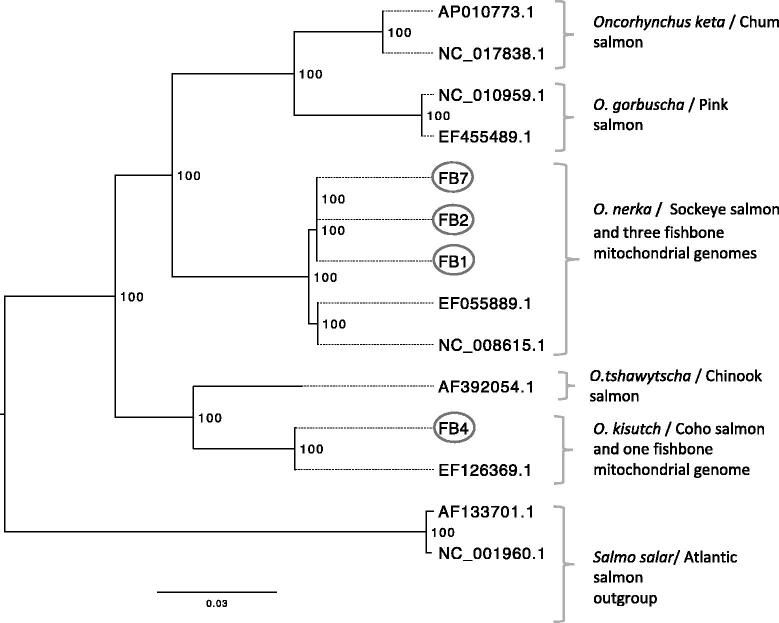
A maximum likelihood phylogeny of four ancient fish mitochondrial genomes and 10 previously reported mitochondrial reference genomes from all North American salmon species (labels correspond to GenBank accession numbers) showed that three of the ancient fish bones group with sockeye salmon and a fourth fishbone groups with coho salmon. Bootstrap support is indicated to the right of each node; tree is midpoint rooted; scale bar represents genetic change as the mean number of nucleotide substitutions per site. GenBank accession numbers for the ancient fish bones: FB1 MG993162, FB2 MH003639, FB4 MH003640, and FB7 MH003641.

The species identifications are consistent with interpretations of a salmon-intensive subsistence strategy of the Riverine Kachemak tradition targeting sockeye salmon and including other species such as coho salmon (Reger 1998).
